# The Greek-Orthodox version of the Brief Religious Coping (B-RCOPE) instrument: psychometric properties in three samples and associations with mental disorders, suicidality, illness perceptions, and quality of life

**DOI:** 10.1186/s12991-017-0136-4

**Published:** 2017-02-16

**Authors:** Vassiliki Paika, Elias Andreoulakis, Elisavet Ntountoulaki, Dimitra Papaioannou, Konstantinos Kotsis, Vassiliki Siafaka, Konstantinos N. Fountoulakis, Kenneth I. Pargament, Andre F. Carvalho, Thomas Hyphantis, On behalf of the ASSERT-DEP Study Group members

**Affiliations:** 1 Department of Psychiatry, Faculty of Medicine, School of Health Sciences, University of Ioannina, 45110 Ioannina, Greece; 2 Third Department of Psychiatry, School of Medicine, Aristotle University of Thessaloniki, Thessaloniki, Greece; 3 Department of Speech and Language Therapy, Technological Educational Institute of Epirus, Ioannina, Greece; 4 Department of Psychology, Bowling Green State University, Bowling Green, OH USA; 5 Department of Clinical Medicine and Translational Psychiatry Research Group, Faculty of Medicine, Federal University of Ceará, Fortaleza, CE Brazil

**Keywords:** Religious coping, Religiousness, Mental disorder, Depression, Anxiety, Suicidal risk, Illness perceptions, Quality of life, Psychometric properties, Chronic illness

## Abstract

**Background:**

The B-RCOPE is a brief measure assessing religious coping. We aimed to assess the psychometric properties of its Greek version in people with and without long-term conditions (LTCs). Associations between religious coping and mental illness, suicidality, illness perceptions, and quality of life were also investigated.

**Methods:**

The B-RCOPE was administered to 351 patients with diabetes, chronic pulmonary obstructive disease (COPD), and rheumatic diseases attending either the emergency department (*N* = 74) or specialty clinics (*N* = 302) and 127 people without LTCs. Diagnosis of mental disorders was established by the MINI. Associations with depressive symptom severity (PHQ-9), suicidal risk (RASS), illness perceptions (B-IPQ), and health-related quality of life (WHOQOL-BREF) were also investigated.

**Results:**

The Greek version of B-RCOPE showed a coherent two-dimensional factor structure with remarkable stability across the three samples corresponding to the positive (PRC) and negative (NRC) religious coping dimensions. Cronbach’s alphas were 0.91–0.96 and 0.77–0.92 for the PRC and NRC dimensions, respectively. Furthermore, NRC was associated with poorer mental health, greater depressive symptom severity and suicidality, and impaired HRQoL. In patients with LTCs, PRC correlated with lower perceived illness timeline, while NRC was associated with greater perceived illness consequences, lower perceived treatment control, greater illness concern, and lower illness comprehensibility.

**Conclusions:**

These findings indicate that the Greek-Orthodox B-RCOPE version may reliably assess religious coping. In addition, negative religious coping (i.e., religious struggle) is associated with adverse illness perceptions, and thus may detrimentally impact adaptation to medical illness. These findings deserve replication in prospective studies.

## Background

A large body of evidence indicates that individuals may rely on religiousness to cope with adverse life events [1, 2]. In addition, religious coping has emerged as a relevant construct that may influence adaptation to adverse life events and stressors, including physical illnesses [1–3]. Individuals coping with adversity including chronic physical illnesses, also called long-term conditions (LCTs), may use both positive and negative religious coping strategies, which may influence adaptation processes in opposing manners [3–5].

The Brief Religious Coping (B-RCOPE) [5, 6] has been largely used to assess religious coping. This instrument developed by Pargament and colleagues, and has been translated and validated in a number of languages including Arab [7], Iranian [8], Polish [9], and Spanish [10] as well as in several religions and doctrines including Protestants and Catholics [6], Hindus [11], and Jewish [12]. According to Pargament’s theory of religious coping, religious coping refers to the way one may understand and overcome stressful life situations using approaches related to the sacred [13]. The term “sacred” in this theory refers not only to traditional notions of God, holiness or higher powers, but also to other aspects of life that are related to the divine [14] and includes a broad range of cognitive, behavioral, and interpersonal responses to stressors [15]. The B-RCOPE is an abridged 14-item version of the full-length 63-item RCOPE scale and it similarly captures positive and negative religious coping dimensions [13]. Positive religious coping (PRC) comprises strategies that may lead to beneficial adaptation, and includes seeking God’s love, protection or forgiveness, stronger connection with a transcendent power, praying for others, and reappraisal of the stressor as a benefit. On the contrary, negative religious coping (NRC), also referred to as “religious/spiritual struggle,” encompasses doubt and strain around sacred matters with the divine, questioning God’s existence, doubting God’s love, and redefining the stressor as God’s punishment or as an act of an evil power [13].

The B-RCOPE has been administered to several populations including adolescents [9], college students [10], patients undergoing cardiac surgery [16], cancer patients and their caregivers [17, 18], and medically ill elderly hospitalized patients [19], among others. Overall, PRC has been associated with favorable outcomes after exposure to stressful events [19] including but not limited to better overall quality of life and less symptoms of psychological distress (e.g., anxiety and depression). Conversely, NRC has been associated with deleterious outcomes [18–20]. Studies have also shown that, in individuals coping with long-term conditions, religious coping may influence mental health, health-related quality of life (HRQoL), treatment adherence, or even survival [20–23].

On the other hand, in medical illness, little attention has been given to the relationship between religious coping and illness perceptions. Patients develop representations of their illness to make sense of and respond to their illness’s adversities [24], and each patient has his/her own ideas about the *identity*, *cause*, *timeline,* or the *consequences* of the illness as well as beliefs about the *cure* and *controllability* of the disease. These illness perceptions shape the patients’ attitudes and emotional responses towards their illness and its treatment [24]. Moreover, accumulating evidence indicates that illness perceptions may be relevant predictors of outcomes in patients with LTCs [25]. Scarce studies reported that people active in faith/church exhibited more adaptive illness perceptions [26], and in patients with chronic kidney disease, aspects of religious coping have been found to mediate the relationship between illness perceptions and HRQoL [27]. However, the influence of religious coping on medical patients’ illness perceptions deserves further investigation.

The aims of the present study were (1) to assess the factorial structure of a Greek version of B-RCOPE and its stability in 3 different populations (i.e., healthy participants, patients with LTCs attending the emergency department (ED), and patients with LTCs attending speciality clinics; (2) to evaluate its internal consistency; and (3) to test the concurrent and convergent validity of the instrument analyzing the independent associations of PRC and religious struggle with mental disorders, suicidality, and HRQoL. A secondary aim was to explore the relationship of B-RCOPE dimensions with specific illness perceptions of patients with LTCs as measured with the Brief Illness Perceptions Questionnaire [28].

## Methods

### Participants

Data were collected during the baseline assessment of the cohort study “Assessing and enhancing resilience to depression in people with long term medical conditions in the era of the current Greek social and financial crisis.” Its main objective is to develop psychosocial strategies to enhance resilience to depression in vulnerable patients with LTCs affected by the current Greek social and financial crisis, through a program of applied clinical research.

A total of 505 participants took part in this study. The sample comprised 376 patients with LTCs and 129 participants without LTCs. The patient sample consisted of patients with at least one of three LTCs: type-II diabetes mellitus (DM), rheumatological disorders (RD), and chronic pulmonary obstructive disease (COPD) who were seeking unscheduled or urgent care at the ED of the University Hospital of Ioannina (*N* = 74) or were attending routine care in the respective follow-up specialty clinic (*N* = 302) during a 6-month period (9/2015–3/2016). Exclusion criteria were inability to read and write Greek, mental retardation, active psychosis, state of intoxication or confusion, or too severely unwell physically.

Of the 116 patients in the ED who were approached, 86 were eligible and 74 agreed to participate (response rate 86.1%): 33 with DM only, 5 with RD only, 22 with COPD only, and 14 with a combination of conditions. Ages ranged from 18 to 94 years old (mean, 66.2; SD, 14.7), 43 males (58.1%) and 31 females (41.9%). Seven ED patients (9.4%) did not complete the B-RCOPE and were thus excluded from the current study. Οf the 360 patients attending specialty clinics who were approached, 350 were eligible and 302 agreed to participate (response rate 86.3%): 88 with DM only, 172 with RD only, 7 with COPD only, and 35 with a combination of conditions. Ages ranged from 20 to 88 years old (mean, 59.4; SD, 14.0), 157 males (52.0%) and 145 females (48.0%). Eighteen patients (5.9%) did not complete the B-RCOPE and were excluded from this study.

People without LTCs were recruited from the hospital staff. Healthcare workers in all hospital’s departments and clinical units were invited to participate. Exclusion criterion was a self-reported LTC (i.e., DM, RD, or COPD). Two hundred and twenty potential participants were approached, 200 were eligible and 129 agreed to participate (response rate 64.5%). Ages ranged from 20 to 58 years old (mean, 39.5; SD, 10.7), 32 males (24.8%) and 97 females (75.2%). Two participants (1.5%) did not complete the B-RCOPE and were excluded from the present study. No statistically significant differences were found in age, gender, education, and marital status between participants and non-participants as well as between those who completed B-RCOPE and those who did not provide complete responses to this instrument across all samples (data available upon request).

Researchers were in the hospital from 8.00 am to 4.00 pm every day and participants were consecutively recruited during this time frame. Participants of either gender aged ≥18 years old were considered for inclusion and, for patients, a diagnosis of DM, RD, or COPD was confirmed by the attending physician. Three trained research psychologists (EN, VP, DP) collected the data. The interviewers had at least 4 years of research and clinical experience at the Department of Psychiatry of the University of Ioannina and were also trained on the administration of diagnostic instruments and screens. The interviewers were blind to scores of the self-report questionnaires, which were administered on the same day. All study procedures were in accordance with the World Medical Association Helsinki Declaration. The study was approved by the hospital’s ethics committee (617/17–09-2015). Signed informed consent was obtained from all participants.

### Measures and study instruments

Socio-demographic variables including age, gender, marital status, residence, educational level, employment status, and occupation were collected for all participants. Information regarding religious affiliations and levels of religious participation was obtained using the Duke University Religion Index (DUREL) [29]. DUREL is a 5-item Likert-type scale measuring three dimensions of religiosity: organizational religious activity (ORA), non-organizational religious activity (NORA), and intrinsic religiosity (IR), with scores ranging from 1 to 5 for IR and from 1 to 6 for ORA and NORA. For patients with LTCs, clinical features, disease severity indices, and laboratory data were obtained from hospital records. Coexisting medical diseases were scored using the Charlson comorbidity scale [30], which is one of the most extensively used comorbidity indices.

*Religious coping* was assessed with the Brief Religious Coping inventory (B-RCOPE). The B-RCOPE comprises 14 items distinguishing between Positive Religious Coping (PRC) and Negative Religious Coping (NRC) styles: 7 items reflect PRC and 7 items reflect NRC [15]. The score of each item ranges from 1 (‘not at all’) to 4 (‘a great deal’), and the total score ranges from 7 to 28 for each subscale; the higher the score, the stronger the PRC and NRC, respectively. PRC items rely on a secure relationship with God, whereas NRC items reflect religious struggle that grows out of a more tenuous relationship with God [31]. Evidence indicates higher means and greater variance for the PRC than for the NRC subscales, and numerous studies support the validity and reliability of the B-RCOPE [5]. The B-RCOPE was translated from English into Greek with Prof. Pargament’s written permission, with unanimous consensus by a bilingual group of 3 psychiatrists and a clinical psychologist, using the back-translation method [32, 33]. The Greek and the original versions of the questionnaire are displayed in "Appendix".

Diagnoses of mental disorder were established using the Greek version 5.0.0 of the Mini International Neuropsychiatric Interview (MINI) [34]. The MINI is a structured psychiatric interview that ascertains the diagnosis of mental disorders according to DSM-IV or ICD-10 criteria [35]. It focuses mainly on current diagnosis and contains 120 questions for screening 17 axis I DSM-IV disorders. MINI has been previously used in studies with Greek medical patients [36–38].

*Depressive symptom severity* was assessed using the validated Greek version of the Patient Health Questionnaire-9 (PHQ-9) [36, 39]. This instrument screens for DSM-IV major depressive disorder. The frequency of symptoms is rated over the past 2 weeks on a 0–3 Likert-type scale; summed scores range from 0 to 27. Higher scores indicate more severe symptoms. Cronbach’s alpha for the PHQ-9 in this sample was 0.83.

*Suicidal risk* was assessed using the standardized Greek version of the Risk Assessment Suicidality Scale (RASS) [40]. RASS is a 12-item self-report instrument of suicidal risk behaviors which contains items relevant to intention, life, and history of suicide attempts. Items are rated on a 0–3 Likert-type scale (*not at all* to *very much*) and the scores were transformed in accordance to suggestions of the standardization study for use within the Greek population [40]. In patients with LTCs attending the ED, Cronbach’s alpha for the RASS was 0.80 [38]. Higher scores indicate greater suicidal risk.

*Illness perceptions* were assessed using the Brief Illness Perception Questionnaire (B-IPQ) [28]. The B-IPQ is a nine-item scale developed to assess the cognitive and emotional representations of illness using a single-item approach on a 0–10 scale to assess perceptions relevant to: *consequences* (how much does your illness affect your life?), *timeline* (how long do you think your illness will continue?), *personal control* (how much control do you feel you have over your illness?), *treatment control* (how much do you think your treatment can help your illness?), *identity* (how much do you experience symptoms from your illness?), *concern* (how concerned are you about your illness?), *emotions* (how much does your illness affect you emotionally?), and illness *comprehensibility* (how well do you feel you understand your illness?). The B-IPQ is a widely used instrument and a recent systematic review with meta-analysis showed that pooled correlations between illness perceptions and depression, anxiety, and quality of life were consistent with previous research and theory [25].

*Health*-*related quality of life* (HRQoL) was assessed using the 26-item validated Greek version of the World Health Organization quality of life instrument, short form (WHOQOL-BREF) [41]. It assesses six domains, overall HRQoL, general health, physical, mental, social relations, and environment HRQoL. Each item is rated on a 5-point Likert scale and the scores are transformed on a scale from 0 to 100. Higher scores indicate better HRQoL.

### Statistical analysis

All analyses except factor analysis for the B-RCOPE scale were performed using the Statistical Package for the Social Sciences (SPSS) version 21.0 (SPSS Inc., Chicago, IL, USA) for Windows. Out of 505 patients, 27 (5.3%) did not complete B-RCOPE questionnaire at all; no significant differences between those who completed and those who did not complete the B-RCOPE. This, along with the low percentages of missing values across the other instruments (MINI: 0/478, PHQ-9: 2/478, RASS: 6/478, WHOQoL-BREF: 5/478, B-IPQ: 0/351) allowed their listwise deletion; the final size of the overall sample was 478. Summary statistics for all variables were calculated. Normality was tested with the Kolmogorov–Smirnov test [42]. Descriptive characteristics of the distribution of B-RCOPE items scores (mean, standard deviation, skewness, and kyrtosis) were calculated and are presented in Table 1 for the entire sample and Table 2 for the other three samples. To test whether the B-RCOPE items gather in clusters according to the original version of the instrument and to assess the stability of its factorial structure across the 3 samples, four separate exploratory factor analyses were performed separately for each sample as well as for the entire sample by means of the FACTOR software [43, 44]. Due to the nature of the B-RCOPE item scores (4-point Likert-type ordinal variables) and the excessive skewness and kyrtosis in their distribution, polychoric correlations were used to construct the correlation matrix [45, 46]. The procedure used for determining the number of dimensions was the optimal ιmplementation of parallel analysis (PA) [47]. Unweighted least squares (ULS) method was used for factor extraction, and weighted varimax rotation with Promin Rotation to maximize factor simplicity was used to produce rotated factor matrices [48]. Internal consistencies (Cronbach alphas) were calculated for the factors derived from exploratory factor analysis and are presented in Table 3. Item-test correlations, i.e., Spearman rho correlation coefficients between each B-RCOPE item and the factors obtained (namely PRC and NRC), were also calculated (Table 4). Finally, the possibility of a “floor” or “ceiling effect” was also investigated (Table 5).

**Table 1:** Descriptive characteristics of B-RCOPE scale items in the entire sample (*N* = 478)

	Mean	SD	Skewness	Kyrtosis
Item 1	2.192	1.093	0.356	−1.216
Item 2	2.552	1.103	−0.072	−1.319
Item 3	2.343	1.123	0.159	−1.361
Item 4	2.092	1.133	0.522	−1.182
Item 5	2.077	1.080	0.528	−1.055
Item 6	2.316	1.139	0.195	−1.389
Item 7	1.912	1.080	0.797	−0.762
Item 8	1.398	0.739	1.844	2.586
Item 9	1.303	0.668	2.355	5.096
Item 10	1.531	0.841	1.485	1.235
Item 11	1.460	0.815	1.681	1.789
Item 12	1.159	0.522	3.645	13.508
Item 13	1.421	0.881	2.006	2.704
Item 14	1.686	0.992	1.167	0.013

**Table 2:** Descriptive characteristics of B−RCOPE scale items in the three separate samples

	Healthy participants (*N* = 127)				ED medical patients (*N* = 67)				Routine care medical patients (*N* = 284)			
	Mean	SD	Skewness	Kyrtosis	Mean	SD	Skewness	Kyrtosis	Mean	SD	Skewness	Kyrtosis
Item 1	1.945	0.994	0.751	−0.529	2.343	1.136	0.050	−1.450	2.268	1.112	0.264	−1.298
Item 2	2.158	1.080	0.449	−1.088	2.731	1.081	−0.327	−1.154	2.687	1.078	−0.235	−1.216
Item 3	2.205	1.108	0.367	−1.227	2.373	1.139	0.165	−1.380	2.398	1.125	0.069	−1.382
Item 4	1.724	1.013	1.136	−0.033	2.239	1.182	0.369	−1.381	2.222	1.139	0.336	−1.324
Item 5	1.858	0.965	0.827	−0.408	2.000	1.059	0.710	−0.739	2.194	1.119	0.358	−1.274
Item 6	2.268	1.094	0.298	−1.223	2.388	1.114	0.189	−1.304	2.320	1.168	0.158	−1.472
Item 7	1.693	0.955	1.208	0.336	2.149	1.184	0.549	−1.226	1.954	1.094	0.695	−0.948
Item 8	1.449	0.742	1.776	2.858	1.328	0.705	2.113	3.576	1.391	0.746	1.843	2.430
Item 9	1.260	0.566	2.363	5.716	1.224	0.623	3.291	11.307	1.342	0.718	2.156	3.906
Item 10	1.528	0.834	1.579	1.726	1.433	0.763	1.814	2.727	1.556	0.862	1.393	0.866
Item 11	1.394	0.747	1.996	3.423	1.493	0.877	1.761	2.120	1.482	0.830	1.551	1.236
Item 12	1.134	0.510	4.237	18.423	1.254	0.636	2.648	6.620	1.148	0.497	3.781	14.876
Item 13	1.158	0.478	3.523	13.672	1.567	0.988	1.556	1.046	1.504	0.968	1.722	1.482
Item 14	1.559	0.823	1.241	0.430	1.567	0.891	1.379	0.747	1.771	1.073	1.037	−0.413

**Table 3:** Factor analysis of the Greek version of the Brief-RCOPE in the entire sample and across the three different samples

	Entire sample (*N* = 478)		Healthy participants (*N* = 127)		ED medical patients (*N* = 67)		Routine care medical patients (*N* = 284)	
	F1	F2	F1	F2	F1	F2	F1	F2
Item 1	*0.909*	−0.139	*0.840*	−0.113	*0.817*	−0.021	*0.886*	−0.149
Item 2	*0.971*	−0.083	*0.907*	−0.066	*0.865*	−0.124	*0.961*	−0.082
Item 3	*0.849*	−0.024	*0.680*	0.102	*0.792*	−0.070	*0.876*	−0.065
Item 4	*0.928*	−0.112	*0.895*	−0.166	*0.759*	−0.130	*0.890*	−0.050
Item 5	*0.640*	0.235	*0.587*	0.246	*0.674*	0.159	*0.570*	0.230
Item 6	*0.574*	0.096	*0.643*	0.158	*0.317*	0.217	*0.611*	0.028
Item 7	*0.907*	−0.095	*0.865*	−0.124	*0.778*	0.019	*0.845*	−0.065
Item 8	−0.112	*0.880*	−0.100	*0.875*	0.003	*0.622*	−0.155	*0.829*
Item 9	−0.047	*0.838*	0.043	*0.480*	−0.021	*0.539*	−0.134	*0.966*
Item 10	−0.066	*0.901*	−0.158	*0.857*	0.007	*0.683*	−0.070	*0.868*
Item 11	0.095	*0.711*	0.071	*0.565*	−0.010	*0.605*	0.127	*0.630*
Item 12	0.048	*0.696*	0.070	*0.459*	0.040	*0.503*	0.035	*0.553*
Item 13 (demonic reappraisal)	*0.438*	0.195	0.239	0.253	*0.368*	0.192	*0.391*	0.222
Item 14	−0.003	*0.519*	−0.043	*0.408*	−0.021	*0.476*	0.009	*0.536*
Eigenvalues	6.491	2.814	5.658	2.090	4.786	2.321	6.177	2.625
Variance explained (%) based on eigenvalues	46.36	20.10	40.41	14.93	34.19	16.58	44.12	18.75
Reliability of rotated factors (Cronbach’s alphas)	0.957	0.917	0.927	0.858	0.909	0.772	0.948	0.919

Factor loadings above 0.3 are presented in italics

**Table 4:** Item-test correlations

	Entire sample (*N* = 478)		Healthy participants (*N* = 127)		ED medical patients (*N* = 67)		Routine care medical patients (*N* = 284)	
	PRCOPE (items 1–7)	NRCOPE (items 8–14)	PRCOPE (items 1–7)	NRCOPE (items 8–14)	PRCOPE (items 1–7)	NRCOPE (items 8–14)	PRCOPE (items 1–7)	NRCOPE (items 8–14)
Item 1	0.832***	0.271***	0.804***	0.281***	0.878***	0.342**	0.817***	0.241***
Item 2	0.890***	0.354***	0.894***	0.330***	0.880***	0.292*	0.875***	0.357***
Item 3	0.841***	0.339***	0.816***	0.343***	0.833***	0.240	0.848***	0.349***
Item 4	0.842***	0.308***	0.793***	0.226***	0.800***	0.179	0.856***	0.359***
Item 5	0.757***	0.451***	0.784***	0.506***	0.799***	0.401***	0.729***	0.440***
Item 6	0.694***	0.302***	0.839***	0.452***	0.461***	0.268*	0.698***	0.253***
Item 7	0.819***	0.319***	0.794***	0.245**	0.882***	0.353**	0.806***	0.331***
Item 8	0.211***	0.655***	0.336***	0.766***	0.194	0.648***	0.179**	0.629***
Item 9	0.228***	0.579***	0.234**	0.527***	0.096	0.495***	0.250***	0.626***
Item 10	0.270***	0.733***	0.269**	0.743***	0.196	0.689***	0.286***	0.740***
Item 11	0.313***	0.703***	0.348***	0.666***	0.110	0.704***	0.335***	0.718***
Item 12	0.245***	0.432***	0.301***	0.435***	0.192	0.487***	0.221***	0.421***
Item 13 (demonic reappraisal)	0.416***	0.513***	0.334***	0.352***	0.443***	0.536***	0.411***	0.554***
Item 14	0.151***	0.683***	0.124	0.628***	0.016	0.634***	0.175**	0.713***

Spearman Rho correlation coefficients

*** *p* < 0.001, ** *p* < 0.01, * *p* < 0.05

**Table 5:** Investigation for a possible « floor » or « ceiling effect » in B-RCOPE scale

	Entire sample (*N* = 478)		Healthy participants (*N* = 127)		ED medical patients (*N* = 67)		Routine care medical patients (*N* = 284)	
	PRC (items 1–7)	NRC (items 8–14)	PRC (items 1–7)	NRC (items 8–14)	PRC (items 1–7)	NRC (items 8–14)	PRC (items 1–7)	NRC (items 8–14)
Minimum score	7	7	7	7	7	7	7	7
Percentage of cases with minimum score	12.3%	38.1%	15.7%	37.0%	4.5%	37.3%	12.7%	38.7%
Maximum score	28	25	28	23	27	24	28	25
Percentage of cases with maximum score	2.7%	0.2%	3.9%	0.8%	1.5%	1.5%	2.8%	0.4%

*PRC* Positive Religious Coping, *NRC* Negative Religious Coping

The criterion and concurrent validity was tested with the following hypotheses in mind: (a) PRC is mostly associated with measures of positive psychological constructs and NRC is tied to signs of poorer mental health [5]. Accordingly, B-RCOPE dimensions should be associated with a diagnosis of mental disorder, especially as far as NCR is concerned. For this, two-tailed t-tests were performed to assess the differences between those diagnosed with mental disorder and those who did not in PRC/NRC scores. To quantify the differences, simple logistic regression analyses were next performed with dependent variable the specific mental diagnosis and independent variable the PRC or NCR scores. In addition, to assess the relationship of PRC/NRC with depressive symptom severity as assessed with the PHQ-9 and suicidal risk as assessed with the RASS, bivariate correlation analyses were performed followed by partial correlation analyses adjusted for age, sex, education, family status, disease type, and comorbidities. (b) PRC is significantly and positively correlated with well-being and NRC is negatively associated with constructs representing well-being [5]. Accordingly, PRC scores should be positively associated with HRQoL scores and the opposite should occur for the NRC scores. To test this, bivariate correlation analyses were performed followed by partial correlation analyses adjusted for age, sex, education, family status, disease type, and comorbidities. (c) B-RCOPE dimensions are associated with coping processes, with PRC being positively associated with behavior and active coping [49, 50] and NCR with anger and avoidant coping [49]. Since medical illness patients’ own views and beliefs about their condition (i.e., illness perceptions) can influence their way of coping and responding both emotionally and physically to their illness [51], we assumed that B-RCOPE dimensions are associated with the patients’ illness perceptions. As no studies have assessed the relationship of B-IPQ with B-RCOPE, no clear hypotheses were adopted and the analyses were exploratory. For this, bivariate correlation analyses were performed followed by partial correlation analyses adjusted for age, sex, education, family status, disease type, and comorbidities.

## Results

### Religious affiliations and levels of religious participation

All participants declare a Greek-Orthodox religion; 170 (35.5%) declared in DUREL attend church or other religious meetings up to few times a month, 276 (57.8%) once or a few times a year, and 32 (6.7%) never. In addition, 243 (50.8%) responded they spend time in private religious activities, such as prayer, meditation, or Bible study daily or up to once a week, 57 (11.9%) a few times a month, and 178 (37.3%) rarely or never. There were no statistically significant differences across the three samples in *organizational religious activity* and *intrinsic religiosity* as assessed with DUREL, after adjustment for age and gender. However, the patient groups, either ED patients (*p* = 0.032) or patients in specialty clinics (*p* < 0.001), reported spending more time in private religious activities (*non*-*organizational religious activity*) compared to participants without LTCs (*F*_[2,461]_ = 8.14, *p* < 0.001).

### Factor structure

**Fig. 1:**
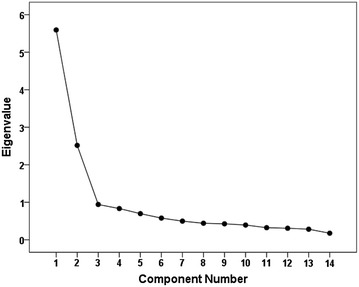
*Scree plot* of the eigenvalues of the B-RCOPE scores

Four independent exploratory factor analyses were performed for the total sample and for each one group of participants, i.e., the healthy participant sample, the attending the ED medical patient sample, and the routine care medical patient sample. Adequacy of correlation matrices was verified across all samples. Kaiser-Meyer-Olkin test yielded values of 0.905, 0.880, 0.791, and 0.890 respectively; all Bartlett’s tests were significant for sphericity (*x* ^2^ = 3398.9, df = 91, *p* < 0.001; *x* ^2^ = 1043.2, df = 91, *p* < 0.001; *x* ^2^ = 577.2, df = 91, *p* < 0.001; and *x* ^2^ = 2051.9, df = 91, *p* < 0.001, respectively), supporting the factorability of the correlation matrices. An inspection of the scree plots in all groups revealed two large components with the most prominent “elbow” occurring after the 2nd component, followed by minor “elbows” reflecting small eigenvalues thereafter (Fig. 1). Based on this and on parallel analysis (PA) [47], it was concluded that a two-factor solution best fitted the results. All rotated solutions confirmed the presence of a coherent two-dimensional structure. In the total sample, the first factor explained 46.6% and the second 20.1% of the variance, summing up to 66.7% (Table 3). Inter-factor correlation coefficients were 0.44, 0.51, 0.33, and 0.47 for the entire sample and the other three samples, respectively.

Factor loadings for all samples are presented in Table 3. Factor 1 was loaded saliently by items 1–7 with item loadings generally greater than 0.60; this factors is relevant to the “positive religious coping” dimension of the original version. It was concluded that this factor represents the “Positive Religious Coping” dimension of B-RCOPE. Of note, item 13 (demonic reappraisal) presented medium secondary loadings on factor 1, ranging from 0.239 to 0.438.

Factor 2 was loaded saliently by items 8-14 with item loadings generally higher than 0.50; this factor is relevant to the “negative religious coping” dimension of the original version, with the exception of item 13 (demonic reappraisal). This item showed relatively lower loadings on factor 2 compared to factor 1 in all samples except for ED medical patients where it presented a higher loading on factor 2, but still relatively low loadings on both factors. Nevertheless, as shown in Table 4, item 13 demonstrated higher correlation coefficients with factor 2 compared to factor 1 in all four samples. In addition, when we re-run the same analyses without including item 13, we observed similar item loadings in factors 1 and 2 (data available upon request). As “demonic reappraisal” could be used either as part of the negative religious coping dimension or could stand by its own as a separate indicator [52], it was concluded that the second factor represents the “Negative Religious Coping” dimension of B-RCOPE.

### Internal consistency

The Greek version of the B-RCOPE showed adequate internal consistency. Cronbach’s alpha coefficients were 0.91–0.96 for the PRC dimension, 0.77–0.92 for the NRC dimension across the three groups, and 0.96 and 0.92 for the total sample, respectively (Table 3). Since “demonic reappraisal” presented low loading on factor 2, we computed alpha coefficient for factor 2 after deleting this item. Only trivial improvement of alphas was observed in all samples (data available upon request).

### “Floor” or “ceiling effect”

No “ceiling effect” was observed concerning both PRC and NRC dimensions, as the percentage of cases that achieved the maximum score ranged across samples from 1.5–3.9% to 0.2–1.5%, respectively. PRC marginally demonstrated a possibility for a “floor effect” in the healthy participant sample, where the percentage of cases that achieved the minimum score was 15.7%. In all other samples, however, the relevant percentage ranged from 4.5 to 12.7%. On the contrary, NRC consistently demonstrated a considerable possibility for a “floor” effect, as the percentage of cases that achieved the minimum score was above 37% in all samples (Table 5).

### Religious coping and mental illness

One hundred and ninety-three (40.4%) participants were diagnosed with a mental disorder, 98 (20.5%) with major depressive disorder, 74 (15.5%) with generalized anxiety disorder, and 27 (5.6%) with panic disorder. As shown in Table 6, people diagnosed with any mental illness, either with major depressive disorder, panic disorder, or generalized anxiety disorder presented higher scores in both PRC and NRC compared to those without a mental disorder. The binary logistic regression analyses performed to quantify the differences confirmed these associations and revealed slightly stronger associations of NRC with mental illness compared to those of the PRC (Table 6).

**Table 6:** Religious coping and mental Illness in the total sample (*N* = 478)

	Any mental illness		Major depression		Panic disorder		Generalized anxiety disorder	
	Yes (*N* = 193)	No (*N* = 285)	Yes (*N* = 98)	No (*N* = 285)	Yes (*N* = 27)	No (*N* = 285)	Yes (*N* = 74)	No (*N* = 285)
Positive Religious coping								
Mean ± SD (Two-tailed t tests)	16.5 ± 6.5	14.8 ± 6.1***	17.3 ± 6.5	15.0 ± 6.2***	19.9 ± 5.9	15.2 ± 6.2***	16.9 ± 6.1	15.2 ± 6.3*
Odds ratios (95% CI)^a^	1.04 (1.014–1.075)**		1.07 (1.028–1.108)***		1.13 (1.056–1.205)***		1.06 (1.014–1.103)**	
Negative Religious coping								
Mean ± SD (Two-tailed t tests)	10.9 ± 4.4	9.3 ± 3.0***	11.0 ± 4.4	9.7 ± 3.5***	13.4 ± 5.2	9.8 ± 3.5***	11.3 ± 4.6	9.7 ± 3.5***
Odds ratios (95% CI)^a^	1.12 (1.062–1.117)***		1.13 (1.063–1.204)***		1.19 (1.104–1.292)***		1.15 (1.077–1.234)***	

*CI* confidence intervals

*** *p* < 0.001, ** *p* < 0.01, * *p* < 0.05

^a^Binary logistic regression analysis

### Religious coping and depressive symptom severity, suicidal risk, and HRQoL

Table 7 presents the results of the unadjusted and adjusted correlation analyses performed to assess the associations of B-RCOPE with PHQ-9, RASS, and WHOQOL-BREF scores. As shown in this table, PRC was most closely associated with depressive symptom severity, while all other initial associations with the other indices rendered non-significant after covariates were taken into account. On the contrary, all the respective scores remained significant in their relationship with NRC: NRC was most closely associated with depressive symptom severity and suicidal risk, and the greater the NRC the lower the overall HRQoL, satisfaction with general health, as well as physical, mental, social relations, and environment HRQoL. Finally, “demonic reappraisal” was most closely associated with depressive symptom severity and lower social relations HRQoL.

**Table 7:** Religious coping and depressive symptom severity (PHQ-9), suicidal risk (RASS), and health-related quality of life (HRQoL–WHOQOL-BREF) (*N* = 478)

	Positive Religious Coping		Negative Religious Coping		Demonic reappraisal	
	Unadj.	Adj.	Unadj.	Adj.	Unadj.	Adj.
Depressive symptoms	0.196***	0.123**	0.292***	0.252***	0.166***	0.111*
Suicidal risk	0.142**	0.052	0.259***	0.202***	0.100**	0.024
HRQoL						
Overall HRQoL	0.085	0.041	−0.136**	−0.103*	−0.044	−0.043
Satisfaction with general health	0.147***	0.013	−0.140***	−0.114**	−0.144***	−0.017
Physical HRQoL	0.228***	0.069	−0.194***	−0.147***	−0.201***	−0.043
Mental HRQoL	0.165***	0.037	−0.158***	−0.103*	−0.082	−0.030
Social relations HRQoL	0.089*	0.073	−0.152***	−0.108*	−0.203***	−0.103**
Environment HRQoL	0.126**	0.080	−0.267***	−0.243***	−0.086	−0.073

Values shown are bivariate Pearson correlation coefficients (Unadjusted—Unadj.) and partial Pearson correlation coefficients adjusted for age, sex, education, family status, disease type, and comorbidities (Adj.)

*** *p* < 0.001, ** *p* < 0.01, * *p* < 0.05

### Religious coping and illness perceptions (patient sample only)

Table 8 presents the results of the unadjusted and adjusted correlation analyses performed to assess the associations of B-RCOPE with illness perceptions as assessed with the B-IPQ. As shown in this table, the greater the PRC the lower the *timeline* of the underlying medical illness. On the other hand, the greater the NRC the greater the perceived *consequences* of the illness, the lower the beliefs that treatment could control the illness (*treatment control*), the greater the perceived bodily symptoms attributed to the illness (*identity*), the greater the *illness concern* and *emotions* arising by the illness and the lower the *comprehensibility* of the illness. Finally, *comprehensibility* was the only illness perception most closely negatively associated with “demonic reappraisal.”

**Table 8:** Religious coping and illness perceptions (patients only, *N* = 351)

	Positive Religious Coping		Negative Religious Coping		Demonic reappraisal	
	Unadj.	Adj.	Unadj.	Adj.	Unadj.	Adj.
Consequences	0.153**	0.064	0.165**	0.120**	0.092*	0.044
Timeline	−0.079	−0.121*	−0.057	−0.053	−0.072	−0.081
Personal control	−0.025	0.007	−0.116*	−0.091	−0.109*	−0.096
Treatment control	−0.131**	−0.100	−0.126*	−0.157***	−0.067	−0.047
Identity	0.128*	0.055	0.142**	0.095*	0.041	0.006
Illness concern	0.107*	0.072	0.223***	0.184***	0.059	0.051
Comprehensibility	−0.024	−0.007	−0.074	−0.090*	−0.114*	−0.102*
Emotions	0.100	0.066	0.158***	0.121**	0.053	0.041

Values shown are bivariate Pearson correlation coefficients (Unadjusted—Unadj.) and partial Pearson correlation coefficients adjusted for age, sex, education, family status, disease, and comorbidities (Adj.)

*** *p* < 0.001, ** *p* < 0.01, * *p* < 0.05

## Discussion

The results of the present study revealed that the Greek version of B-RCOPE showed a coherent two-dimensional factor structure with remarkable stability across the three samples studied. Items of the Greek-Orthodox version of the B-RCOPE exhibited factor loadings similar to the original version of the instrument [6] with adequate internal consistency reliabilities. Most indices of criterion and convergent validity were in the expected direction. In addition, specific illness beliefs and perceptions of patients with LTCs were significantly and independently associated with PRC and NRC dimensions of the B-RCOPE. Our findings support the validity of the B-RCOPE for use within the Greek-Orthodox population and extend its association with illness perceptions in patients with LTCs.

Similar to the results of the original version [5], the present findings confirmed a 2-factor solution for the instrument. The first 7 items, corresponding to the PRC dimension of the original scale, loaded saliently on the first factor and the remaining 7 items loaded to the NRC factor, thus supporting the original factorial structure of this version of B-RCOPE. There was, however, one exception for NRC: “demonic reappraisal” presented lower than anticipated loadings on factor 2. Lower loadings of “demonic reappraisal” in NRC have been previously reported with the Brazilian Portuguese version of B-RCOPE when used in patients with end-stage renal disease on hemodialysis, and the authors decided to use “demonic reappraisal” as an independent variable [52]. However, when we tested the internal consistency of the NRC with and without “demonic reappraisal,” we found that deleting this item from the factor did not improve its internal consistency reliability. In addition, when we run the factor analyses without item 13, we found similar item loadings. We therefore decided to use in subsequent analyses “demonic reappraisal” in both ways, as part of NRC as well as an independent variable.

In line with the results of numerous previous studies performed in various cultures, languages, and religions [5], both dimensions of this version of B-RCOPE demonstrated adequate internal consistency reliabilities. Cronbach’s alphas for the PRC ranged from 0.91 to 0.96 across all samples, while the NRC exhibited slightly lower Cronbach’s alphas (0.78–0.92) with no significant improvement after exclusion of “demonic reappraisal” from the factor. Similar to our findings, a recent systematic review on the psychometric properties of the B-RCOPE reported that Cronbach’s alphas for NRC were generally lower than those for PRC, with median values for the PRC scale being 0.92 and for the NCR being 0.81 [5]. However, it should be taken into consideration that NRC demonstrated a considerable possibility for a “floor effect.” Therefore, longitudinal studies including B-RCOPE should pay attention when using NRC to test long-term outcomes, as the presence of a “floor effect” may reduce the possibility of detecting important changes over time when the test is applied, since it is likely that extreme items are missing in the lower end of NCR scale and the responsiveness is limited because changes could not be measured in these participants [53].

The criterion validity of the B-RCOPE was supported in three ways. First, NRC was strongly associated with poorer mental health and greater depressive symptom burden, in line with the results of all previous studies performed [5, 54, 55]. It is worth mentioning that, apart from major depression, people diagnosed with panic disorder and generalized anxiety disorder presented also higher NRC scores compared to those without a mental disorder. Although some studies have explored the association of religious coping with anxiety symptoms and anxiety disorders [56–58], depression generally has attracted more attention. However, the influence of anxiety on outcomes in the context of LTCs should not be underestimated; studies have shown that anxiety disorders are also independent predictors of worse outcomes, including suicidal ideation or even suicide attempts [59]. On the other hand, we observed that PRC scores were associated with mental disorders and depressive symptom severity. Although PRC is generally considered to be associated with measures of positive psychological constructs (e.g., better well-being), it is occasionally related also to negative constructs such as depression [5]. In addition, as in Greek-Orthodox religion a disease is often perceived as ‘God’s will’ thereby promoting a stoic-prone attitude especially in older people with physical illnesses [60], it is possible that depressed patients with LTCs cope with their somatic disease through the activation of cognitive processes related to PRC in order to alleviate their affective symptomatology.

Religious struggle (i.e., NRC) was significantly associated with suicidal risk even after controlling for confounders. On the contrary, PRC, although initially associated with RASS scores, did not survive multivariable adjustment to potential confounders. In line with the current findings, NRC has been found to be associated with an increased risk of suicidal ideation in adult patients with advanced cancer even after of controlling for a number of covariates [61] and with a higher frequency and intensity of suicidal ideation in people with psychosis [62]. Additionally, studies on religiousness have shown that religious affiliation and religious service attendance may protect against suicide attempts [63]. Third, all indices of well-being as assessed by the WHOQOL-BREF were negatively associated with NCR, in line with results from previous studies [5, 21, 52, 54].

Our main new finding is that dimensions of B-RCOPE are associated with specific illness perceptions in patients with LTCs. PRC was most closely associated with the *timeline* dimension of illness perceptions, indicating that patients may rely on this coping strategy to understand and adapt to their underlying somatic disease and could be more optimistic about the duration of their illness. On the contrary, those who adopt “religious struggle” as a predominant coping strategy, doubting and straining around sacred matters with the divine, are more concerned and perceive more deleterious consequences of their illness, have lower comprehension of their illness, and do not trust treatment efficacy. The only study examining the association of religious coping and illness perceptions was conducted in a Malaysian sample of 274 patients with end-stage renal disease using a modified version of the B-RCOPE. This cross-sectional study found that religious coping mediated the relationship between illness perceptions and physical and mental HRQoL [27]. Our findings provide initial evidence for the development of psychotherapeutic interventions aiming to mitigate religious struggle in patients with LTCs. Psycho-spiritual interventions for patients with LTCs have gained momentum, with promising preliminary findings; there is initial evidence that both dimensions of the B-RCOPE are sensitive to change after treatment [64, 65].

Strengths of our study include the use of the MINI structured interview for establishing a diagnosis of mental disorder, which was conducted on the same day of the administration of the self-report questionnaires. Also, we used well-recognized and standardized instruments for all measures, and we generally followed the operational framework of Pargament et al. [6] with the original version of the B-RCOPE. In addition, we recruited patients with established LTCs with a high response rate (86%). However, some limitations need to be considered. It could be argued that a limitation of our study lies in the composition of the “healthy” participant sample, which was recruited from hospital staff and could not be considered representative of the general Greek population. In addition, although diagnoses of mental disorders were confirmed by a validated structured diagnostic interview, the drawback of using self-report measures for assessing depressive symptom severity, suicidality, HRQoL, and illness perceptions means that we cannot refute the criticism that an underlying response style might have biased our results. Finally, the cross-sectional design of the current study precludes the establishment of firm causal inferences.

## Conclusions

The results of the present study showed that 2 factors were identified for the Greek-Orthodox version of the B-RCOPE. Internal consistencies were adequate and concurrent and convergent validity quite satisfactory. These findings support the applicability of the Greek version of B-RCOPE within the Greek-Orthodox population, and future studies could further explore the relevance of B-RCOPE dimensions with additional scales and outcomes as well as its predictive validity. Greek clinicians should pay attention when assessing coping using the B-RCOPE in people with LTCs, since present findings showed that negative religious coping is associated with adverse illness perceptions and this may have important clinical implications as far as adaptation to medical illness is concerned.

## Appendix

See Table 9.

**Table 9:** The items of the Greek version of the B-RCOPE

Item no.	Original version	Greek version
1	Looked for a stronger connection with God	Αναζήτησα μία πιο δυνατή σύνδεση με το Θεό
2	Sought God’s love and care	Αναζήτησα την αγάπη και τη φροντίδα του Θεού
3	Sought help from God in letting go of my anger	Αναζήτησα βοήθεια από το Θεό για να μου απαλύνει το θυμό
4	Tried to put my plans into action together with God	Προσπάθησα να βάλω τα σχέδια μου σε δράση μαζί με το Θεό
5	Tried to see how God might be trying to strengthen me in this situation	Προσπάθησα να δω πώς ο Θεός ίσως προσπαθεί να με δοκιμάσει σε αυτήν την κατάσταση
6	Asked forgiveness of my sins	Ζήτησα συγχώρεση για τις αμαρτίες μου
7	Focused on religion to stop worrying about my problems	Συγκεντρώθηκα στη θρησκεία μου για να σταματήσω να αγχώνομαι για τα προβλήματα μου.
8	Wondered whether God had abandoned me	Αναρωτήθηκα αν ο Θεός με έχει εγκαταλείψει
9	Felt punished by God for my lack of devotion	Αισθάνθηκα ότι ο Θεός με τιμωρεί για την έλλειψη αφοσίωσής μου
10	Wondered what I did for God to punish me	Αναρωτήθηκα τι έκανα για να με τιμωρεί ο Θεός
11	Questioned God’s love for me	Αναρωτήθηκα αν ο Θεός με αγαπά
12	Wondered whether my church had abandoned me	Αναρωτήθηκα αν η εκκλησία με έχει εγκαταλείψει
13	Decided the devil made this happen	Αποφάσισα ότι ο διάβολος ευθύνεται γι’ αυτό
14	Questioned the power of God	Αναρωτήθηκα για τη δύναμη του Θεού

## Abbreviations

LTCs: long-term conditions
DM: type-II diabetes mellitus
RD: rheumatological disorders
COPD: chronic pulmonary obstructive disease
MDD: major depressive disorder
GAD: generalized anxiety disorder
B-RCOPE: Brief Religious Coping
PRC: Positive Religious Coping
NRC: Negative Religious Coping
MINI: Mini International Neuropsychiatric Interview
PHQ-9: Patient Health Questionnaire-9
RASS: Risk Assessment Suicidality Scale
HRQoL: health-related quality of life
B-IPQ: Brief Illness Perceptions Questionnaire
DUREL: Duke University Religion Index
ORA: organizational religious activity
NORA: non-organizational religious activity
IR: intrinsic religiosity
